# Reverse transcriptase inhibitors diminish systemic proinflammatory responses to bacterial pathogens

**DOI:** 10.1128/mbio.03412-24

**Published:** 2025-01-14

**Authors:** Karthik Hullahalli, Katherine G. Dailey, Ryan Acbay, Masataka Suzuki, George I. Balazs, Matthew K. Waldor

**Affiliations:** 1 Department of Microbiology, Harvard Medical School, Boston, Massachusetts, USA; 2 Division of Infectious Disease, Brigham & Women’s Hospital, Boston, Massachusetts, USA; 3 Broad Institute of MIT and Harvard, Cambridge, Massachusetts, USA; 4 Howard Hughes Medical Institute, Chevy Chase, Maryland, USA; UCLA School of Medicine, Los Angeles, California, USA

**Keywords:** reverse transcriptase inhibitors, inflammation, sepsis

## Abstract

Bacterial infections can induce exuberant immune responses that can damage host tissues. Previously, we demonstrated that systemic *Escherichia coli* infection in mice causes tissue damage in the liver. This liver necrosis is associated with the expression of endogenous retroviruses, chromosomally integrated retroviruses that encode a reverse transcriptase. Furthermore, nucleotide/nucleoside reverse transcriptase inhibitors (NRTIs) completely prevent tissue damage and subsequent bacterial growth within necrotic lesions. Since liver necrosis is linked to heightened systemic inflammatory responses, we hypothesized that NRTIs diminish inflammation caused by *E. coli* infection and may also have broad impacts on the systemic immune response to bacterial pathogens. Here, we tested this hypothesis by characterizing the effects of NRTIs on the innate immune response to bacteria. In the liver, NRTI administration following *E. coli* inoculation reduced the expression of a large repertoire of proinflammatory transcripts. NRTIs also had systemic anti-inflammatory effects, including reducing proinflammatory cytokine levels in serum in response to *E. coli* in different mouse strains. The anti-inflammatory effects of NRTIs were also apparent in response to lipopolysaccharide (LPS) and *Staphylococcus aureus*, suggesting that the molecular mechanisms underlying the immunomodulatory functions of NRTIs are likely conserved across distinct immune signaling pathways. Moreover, in a model of lethal LPS shock, NRTI administration prevented hypothermia and death. Together, our observations reveal that NRTIs can potently impede systemic inflammatory responses during Gram-positive and Gram-negative bacterial infections. Our findings lay the groundwork for further investigation of the therapeutic scope of NRTIs and the mechanisms underlying their anti-inflammatory effects across non-retroviral infectious diseases.

**IMPORTANCE:**

Inflammatory responses are critical for host control of bacterial infection, but excessive inflammation can damage host tissues and lead to sepsis. Understanding how innate immune responses are controlled during infection is important for developing new approaches to dampen excessive inflammation. In previous work, we found that tissue damage caused by excessive inflammatory responses may be driven by endogenous reverse transcriptases. Here we demonstrate that treatment of mice with reverse transcriptase inhibitors leads to broad reductions in systemic proinflammatory responses during bacterial infections and can protect mice from acute death in a lethal model of sepsis. Our findings indicate that uncovering the mechanisms underlying the anti-inflammatory functions of reverse transcriptase inhibitors may lead to new therapeutics for bacterial infectious diseases.

## OBSERVATION

**Fig 1:**
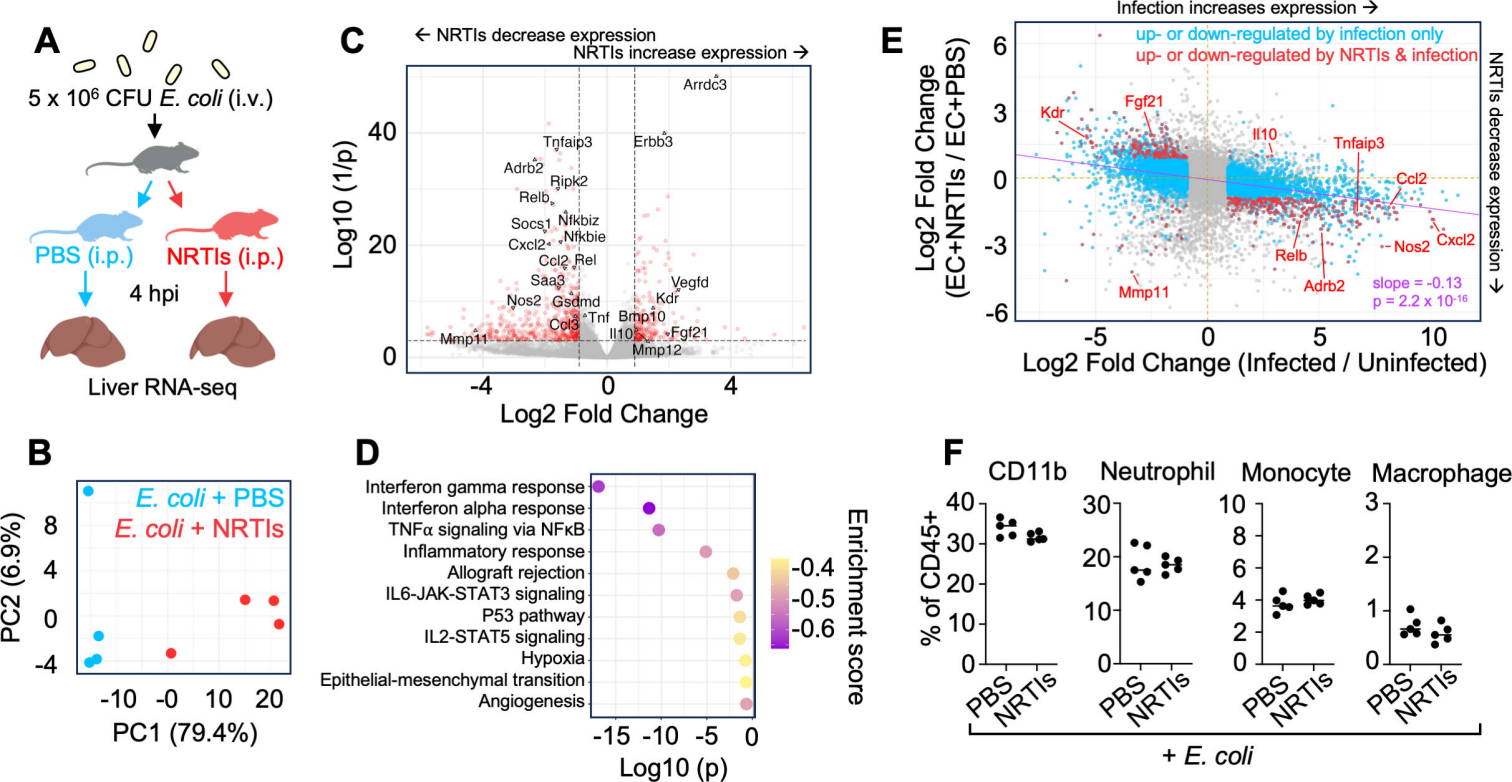
NRTIs diminish proinflammatory gene expression in the liver. (**A**) Schematic of RNA-sequencing experiment. (**B**) PCA plot of hepatic transcriptomes of *E. coli* infected mice at 4 hpi administered NRTIs (red) or PBS (blue). (**C**) Volcano plot of genes differentially expressed in NRTI-treated *E. coli* infected mice compared to PBS-treated *E. coli* infected mice. Red points denote genes with adjusted *P* values less than 0.001 and absolute values of fold changes greater than 0.9. (**D**) Gene set enrichment analysis using fold changes from panel C with Hallmark categories. The top 10 pathways (by adjusted *P* value) are shown. (**E**) Fold changes of genes differentially regulated by *E. coli* infection (*x*-axis) plotted against genes differentially regulated by NRTIs in *E. coli* infected mice. (**F**) Flow cytometry analysis for relative abundance of indicated cell types at 4 hpi in *E. coli* infected mice administered PBS or NRTIs. No statistical significance is observed via one-tailed Student’s *t*-test. For all panels, NRTIs were administered i.p. immediately following i.v. *E. coli* inoculation at 1.6 mg/drug/mouse.

The liver is the major site for the capture of bloodborne bacteria and plays a central role in regulating the systemic inflammatory response to infection (1). We found that certain strains of mice develop liver abscesses following intravenous (i.v.) administration of *E. coli* and that susceptibility to abscess formation correlates with the hepatic expression of endogenous retroviruses (2). Notably, intraperitoneal (i.p.) administration of NRTIs (1.6 mg each of tenofovir disoproxil fumarate, emtricitabine, zidovudine, and abacavir) prevented the liver necrosis associated with *E. coli* infection and led to a marked reduction in bacterial burden (2). Since necrosis is linked to excessive inflammation (3), we tested whether NRTI treatment modulates early hepatic inflammatory responses to *E. coli* infection by characterizing the influence of NRTIs on the liver transcriptome. Female C57BL/6NJ mice, which are highly susceptible to *E. coli* mediated liver abscess formation, were inoculated i.v. with *E. coli* and then administered a single dose of NRTIs or vehicle control i.p. Four hours post inoculation (hpi), the livers were harvested and subject to RNA-sequencing (Fig. 1A).

Principal component analysis demonstrated that mice infected with *E. coli* and administered vehicle control were clearly separated along the first principal component (79.4% of variance) from mice infected with *E. coli* and subsequently administered the NRTI cocktail (Fig. 1B). Differential expression analysis revealed that 221 genes had higher expression (adjusted *P* value < 0.001, log2 fold change >0.9) and 469 genes had lower expression following NRTI treatment in infected mice (Fig. 1C). Several major components of the innate immune response to bacteria had reduced expression following NRTI treatment, including components of the NFkB signaling apparatus (Relb, Rel, Nfkbie, Nfkbiz), numerous proinflammatory cytokines (Tnf, Ccl2, Ccl4, Cxcl2), and other hallmarks of inflammation (Nos2, Myd88, Gsdmd, Saa3). Gene set enrichment analysis (4) corroborated these observations, as inflammatory pathways engaged during bacterial infection were significantly and negatively enriched in NRTI-treated infected mice (Fig. 1D). Transcripts that were more abundant following NRTI administration were primarily associated with growth factor signaling (Vegfd, Fgf21, Bmp10, Kdr).

Our previous RNA sequencing identified hepatic genes induced by *E. coli* infection (2). By intersecting the genes that are differentially regulated by *E. coli* infection and those that are sensitive to NRTIs in infected mice, we assessed how NRTI treatment modulates *E. coli*-induced gene expression in the liver (Fig. 1E). Among the 3,078 genes that are upregulated by *E. coli* infection, 275 (9%) had reduced expression following NRTI treatment, while only 5 (0.16%) had increased expression following NRTI treatment. Among the 2,984 genes that are downregulated by *E. coli* infection, 174 (5.8%) had increased expression following NRTI treatment, while 29 (0.9%) had lower expression following NRTI treatment. Notably, comparing genome-wide fold changes revealed that the effects of NRTI treatment and *E. coli* infection are significantly anti-correlated (slope = −0.13, *P* < 2.2e−16 by Spearman correlation, Fig. 1E). These observations indicate that NRTI treatment partially reverses the hepatic differential gene expression profile resulting from *E. coli* infection, where among the 483 genes differentially regulated by both NRTIs and *E. coli*, 93% are inversely correlated in their expression. In particular, the expression of numerous components of the inflammatory response is diminished in the presence of NRTIs. A notable exception is Il10, a prototypical anti-inflammatory cytokine whose expression increases following *E. coli* infection and increases further following NRTI administration (5, 6).

Rapidly following bloodstream infection, the liver captures bacterial pathogens and recruits inflammatory immune cells, particularly macrophages, monocytes, and neutrophils (1). Therefore, the reduction in inflammatory gene expression in the liver could potentially be attributable to reduced numbers of infiltrating cells. Flow cytometry on mice infected with *E. coli* and administered NRTIs or vehicle control was used to determine whether the number of liver-infiltrating cells is altered following NRTI administration (Fig. 1F). The numbers of monocytes (CD11b^+^, Ly6C^+^, Ly6G^−^), monocyte-derived macrophages (CD11b^hi^, F4/80^+^, Ly6C^−^, Ly6G^−^), and neutrophils (CD11b^+^, Ly6C^+^, Ly6G^+^) relative to total CD45^+^ immune cells were identical in vehicle- and NRTI-treated infected mice. Thus, early after infection, NRTIs appear to diminish inflammatory gene expression in the liver without altering the number of infiltrating cells, potentially accounting for the fact that NRTI treatment does not impair *E. coli* clearance from the liver (2).

**Fig 2:**
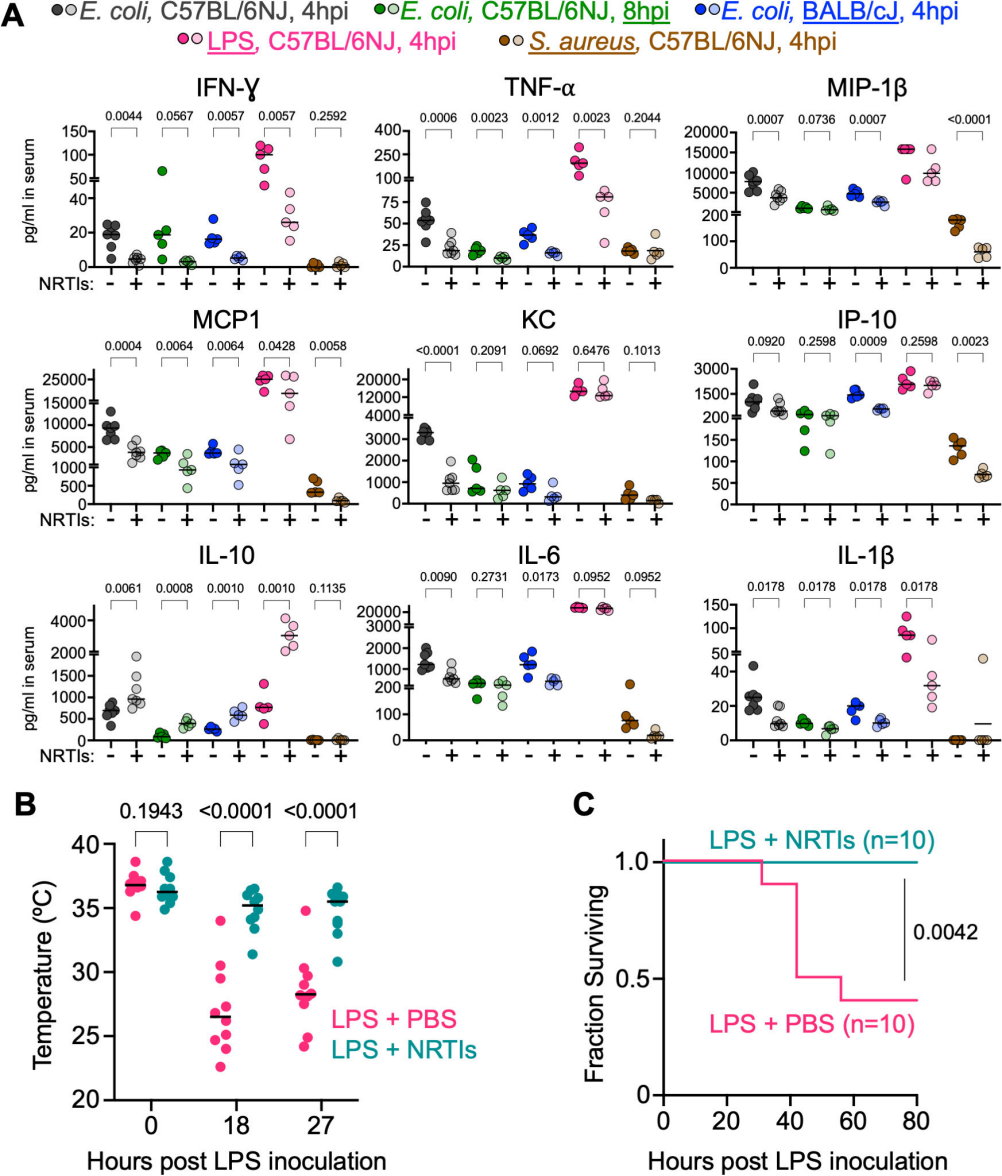
NRTIs diminish systemic proinflammatory responses. (**A**) Cytokine abundances in serum from mice administered various stimuli, across indicated mouse strains and time points, with or without NRTIs. NRTIs were administered i.p. (1.6 mg/drug/mouse) at a single dose immediately following inoculation of indicated stimuli. *P*-values were obtained from Browne-Forsythe and Welch ANOVA tests and corrected for multiple comparisons using the false discovery rate (FDR). *P* value calculations were omitted for conditions where values exceeded limits of detection, and minimum or maximum values are plotted. Axes are modified to visualize all conditions. (**B**) Mice were administered 200 µg of LPS i.v. and either PBS or NRTIs i.p.. *P* values were obtained by multiple *t*-tests followed by FDR correction. (**C**) Survival curves from mice in panel **B**. *P* value was obtained by log-rank test. For panels B and C, NRTIs were administered at 1.6 mg/mouse/drug immediately following inoculation; in addition, NRTIs and vehicle were also administered 3 h prior to LPS inoculation, 3 h post LPS inoculation, and 18 h post LPS inoculation, for a total of 4 doses.

Since the liver plays a central role in regulating immunity across the host, we hypothesized that the anti-inflammatory effects of NRTIs are not solely apparent in the liver. We examined whether these drugs modulate systemic inflammatory responses by profiling the abundance of proinflammatory cytokines in serum (Fig. 2A). In C57BL/6NJ mice infected with *E. coli*, NRTI administration led to significant reductions in several proinflammatory cytokines in serum at 4 hpi (black/gray points, Fig. 2A). This trend continued at 8 hpi (green points), although the amounts of these inflammatory markers were lower at 8 vs 4 hpi, regardless of NRTI treatment. Thus, the ability of NRTIs to blunt inflammatory cytokine levels does not appear to result from a delay their production. Similar reductions were observed at 4 hpi in BALB/cJ mice (blue points), a strain that does not develop liver abscess from *E. coli* infection, indicating that the anti-inflammatory effects of NRTIs are not mouse strain-specific or limited to animals that develop liver necrosis associated with *E. coli* bacteremia. Interestingly, the abundance of IL10 was consistently increased in NRTI-treated mice, corroborating its RNA expression pattern in the liver (Fig. 1C). These results together indicate that NRTIs broadly diminish systemic proinflammatory cytokine production in mice during *E. coli* systemic infection. Notably, since BALB/cJ and C57BL/6NJ are distantly related among inbred mice (7), the observation that NRTIs dampen proinflammatory responses in both strains may suggest that the underlying molecular mechanisms are conserved across mice.

The dominant immunostimulatory molecule on *E. coli* is lipopolysaccharide (LPS) (8), and we tested whether NRTIs modulate the inflammatory response to purified *E. coli* LPS alone. Mice were inoculated with 250 µg of LPS i.v. and administered either NRTIs or PBS control i.p.. NRTI treatment reduced the abundance of most proinflammatory markers that were reduced with *E. coli* infection and increased IL10 production (Fig. 2A, pink points). However, the levels of some cytokines, such as KC and IL-6, that were altered by NRTIs during *E. coli* infection were unchanged during LPS stimulation. We also tested whether NRTIs diminish inflammatory responses induced by the Gram-positive pathogen *Staphylococcus aureus*. Although the stimulation of inflammatory cytokines by *S. aureus* was not as broad or pronounced as observed with *E. coli*, NRTIs significantly reduced the abundance of MCP-1, IP-10, and MIP-1β (Fig. 2A, brown points). Finally, we examined whether NRTI treatment could improve clinical outcomes during lethal LPS shock, a commonly used model for sepsis (9). Mice were administered 200 µg of LPS i.v. (50% lethal dose) and either NRTIs or vehicle control. NRTIs prevented LPS-induced hypothermia (Fig. 2B) and death (Fig. 2C). Together, these data indicate that NRTIs broadly diminish inflammatory responses to bacteria and can protect mice from lethal endotoxin shock. Since NRTIs diminish inflammatory cytokine production in the context of both Gram-negative and Gram-positive infection, the molecular mechanisms underlying the anti-inflammatory effects of NRTIs may be shared between diverse immune signaling pathways.

Several reports in non-infectious contexts and with distinct drug formulations have suggested a role for endogenous retroelements and NRTIs in inflammation (10–12). Some studies have attributed the anti-inflammatory functions of NRTIs to the impedance of nucleic acid signaling of endogenous retroelements through cGAS or off-target inhibition of the NLRP3 inflammasome (13, 14). Whether these pathways are relevant to the anti-inflammatory role of NRTIs in bacterial infection remains an open and important question. Nevertheless, our observations that NRTIs can prevent death from LPS-induced sepsis in mice warrants further studies into whether these drugs or their underlying molecular mechanisms may be relevant in human sepsis, a condition that has defied new effective interventions (15). Critical important questions remain, including understanding how NRTIs modulate infection-triggered innate immune signaling, defining whether these pathways are drug-specific and the determinants of drug potency, and characterizing the therapeutic scope of reverse transcriptase inhibitors for non-retroviral infectious agents.

### Animal experiments

All mice used in this study were 8- to 10-week-old females obtained from The Jackson Laboratories (C57BL/6NJ—#5304 and BALB/cJ—#651). For intravenous inoculation, mice were restrained in a Broome-style restrainer and 100 µL of inoculum was injected into the tail vein with a 27G needle. A heating pad was used to facilitate the dilation of the tail vein. PBS was used as a vehicle for all inocula in this study. NRTIs were prepared from 100 mg/mL stocks in PBS and freshly diluted to 8 mg/mL for each drug (Tenofovir disoproxil fumarate, emtricitabine, zidovudine, and abacavir). Two hundred microliters was administered intraperitoneally. *E. coli* (strain CHS7-STAMP) and *S. aureus* (strain HG003) were prepared by diluting frozen stocks in PBS and were immediately used for inoculation at a dose of 5 × 10^6^ CFU per mouse (16, 17). LPS (Sigma L2630) was stored frozen as 8 mg/mL solutions in PBS and diluted freshly before use. For euthanasia, mice were overdosed with isoflurane and cervical dislocation or exsanguination (for collecting serum) was performed. Body temperature was monitored with a rectal probe.

### Serum collection and cytokine measurements

Blood was collected by cardiac puncture and was left at room temperature for 20 min. Serum was obtained by centrifugation of clotted blood at 1,000 × *g* for 10 min in a refrigerated centrifuge and filtered using a 0.22 µm centrifugal filter. Cytokine abundance was measured using Luminex technology (EveTechnologies) in duplicate.

### RNA-sequencing

Livers were harvested and the right lobe was flash frozen in liquid nitrogen. RNA was extracted with Trizol and the Direct Zol RNA Miniprep Kit (Zymo). Illumina-compatible libraries were prepared with the NEBNext Poly(A) mRNA Magnetic Isolation Module and the NEBNext Ultra II Directional RNA Library Prep Kit for Illumina (New England Biolabs). Libraries were sequenced on a NextSeq 1000 as 1 × 101 nt reads. Reads were processed with TrimGalore and mapped to the reference C57BL/6J chromosome (mm10, GRCm38) using HISAT2 (18). Reads counts were obtained with FeatureCounts and differential expression was performed with DESeq2 (19). Gene set enrichment analysis was performed using the fgsea package in R (20).

### Flow cytometry

Livers were dissected from mice and coarsely chopped with scissors and stored in HBSS + 10 mM EDTA until all mice were dissected. Tissue chunks were washed 3× with 50 mL PBS to remove EDTA and incubated in 10 mL DMEM containing 0.2 mg/mL DNase (Roche 10104159001) and 1 mg/mL collagenase (Sigma-Aldrich C5138) for 15 min at 37°C. Dissociated cells were passed through a 70 µm filter, which was further flushed with ~20 mL of DMEM. Cells were spun at 50 × *g* for 2 min to spin down hepatocytes. The supernatant containing nonparenchymal cells was harvested in a 15 mL conical and centrifuged at 500 × *g* for 5 min. Cells were resuspended in 1 mL of red blood cell lysis buffer (Sigma 11814389001) and incubated for 2 min. Ten milliliters of PBS was added, and cells were centrifuged at 500 × *g* for 5 min. To remove debris, cells were washed three times with PBS + 10 mM EDTA + 2% FBS (FACS buffer) and finally resuspended in 2 mL of FACS buffer. Two hundred microliters of cell suspensions was used for flow cytometry. Cell surface Fc receptors were blocked with anti-CD16/32 (Biolegend 101302) for 5 min and stained with the following antibody cocktail in FACS buffer for 30 min at room temperature; Live/Dead Aqua, anti-Cd45 + Alexa Fluor 700 (BD Bioscience 560510), anti-Ly6c PerCP-Cy5.5 (Biolegend 128011), anti-Ly6g APC-Cy7 (Biolegend 127623), anti-Cd11b APC (Biolegend 553312), anti-Cd11c PE-Cy7 (Biolegend 558079), anti-Cd3e Super Bright 600 (ThermoFisher 63-0031-82), anti-F4/80 PE-Fire 640 (Biolegend 157320), anti-B220 PE-Fire 810 (Biolegend 103287). Cells were washed in FACS buffer twice, fixed in 4% PFA-PBS, and analyzed with the Cytek Northern Lights instrument (Cytek, MA).

### Statistical analyses

Statistical analyses were performed in R or Graphpad Prism. Descriptions of specific methods are provided in the figure legends.

## Data Availability

Raw reads from RNA-Sequencing have been deposited in the Sequencing Read Archive (PRJNA1171823).

## SUPPLEMENTAL MATERIAL

Table S1 mbio.03412-24-s0001.xlsx Count table from RNA-sequencing of mice infected with *E. coli* and administered PBS or NRTIs.

Table S2 mbio.03412-24-s0002.xlsx Complete cytokine measurements from Fig. 2A.

ASM does not own the copyrights to Supplemental Material that may be linked to, or accessed through, an article. The authors have granted ASM a non-exclusive, world-wide license to publish the Supplemental Material files. Please contact the corresponding author directly for reuse.
